# Ac-SDKP decreases mortality and cardiac rupture after acute myocardial infarction

**DOI:** 10.1371/journal.pone.0190300

**Published:** 2018-01-24

**Authors:** Pablo Nakagawa, Cesar A. Romero, Xu Jiang, Martin D’Ambrosio, Ginette Bordcoch, Edward L. Peterson, Pamela Harding, Xiao-Ping Yang, Oscar A. Carretero

**Affiliations:** 1 Hypertension and Vascular Research Division, Department of Internal Medicine, Henry Ford Hospital, Detroit, Michigan, United States of America; 2 Department of Public Health Sciences, Henry Ford Hospital, Detroit, Michigan, United States of America; Escola Paulista de Medicina, BRAZIL

## Abstract

The natural peptide N-Acetyl-Seryl-Aspartyl-Lysyl-Proline (Ac-SDKP) decreases inflammation in chronic diseases such as hypertension and heart failure. However, Ac-SDKP effects on acute inflammatory responses during myocardial infarction (MI) are unknown. During the first 72 hours post-MI, neutrophils, M1 macrophages (pro-inflammatory), and M2 macrophages (pro-resolution) and release of myeloperoxidase (MPO) and matrix metalloproteinases (MMP) are involved in cardiac rupture. We hypothesized that in the acute stage of MI, Ac-SDKP decreases the incidence of cardiac rupture and mortality by preventing immune cell infiltration as well as by decreasing MPO and MMP expression. MI was induced by ligating the left descending coronary artery in C57BL/6 mice. Vehicle or Ac-SDKP (1.6 mg/kg/d) was infused *via* osmotic minipump. Cardiac immune cell infiltration was assessed by flow cytometry, cardiac MPO and MMP levels were measured at 24–48 hrs post-MI. Cardiac rupture and mortality incidence were determined at 7 days post-MI. In infarcted mice, Ac-SDKP significantly decreased cardiac rupture incidence from 51.0% (26 of 51 animals) to 27.3% (12 of 44) and mortality from 56.9% (29 of 51) to 31.8% (14 of 44). Ac-SDKP reduced M1 macrophages in cardiac tissue after MI, without affecting M2 macrophages and neutrophils. Ac-SDKP decreased MMP-9 activation in infarcted hearts with no changes on MPO expression. Ac-SDKP prevents cardiac rupture and decreases mortality post-acute MI. These protective effects of Ac-SDKP are associated with decreased pro-inflammatory M1 macrophage infiltration and MMP-9 activation.

## Introduction

More than 1.5 million people suffer acute myocardial infarction (MI) annually in the United States. About 30% of those patients die within the first 24 hours due to arrhythmias or pump failure. Cardiac rupture is an uncommon but fatal complication in humans. In rodents cardiac rupture is much more common, being between 30 and 60% during the first week post-MI [1]. The structural changes in the extracellular matrix (ECM) during acute inflammation are thought to cause cardiac rupture after myocardial infarction. Matrix metalloproteinases (MMP), a family of zinc-containing endoproteinases released mainly by leukocytes, play a key role in degrading the ECM of the heart [2]. Specifically, MMP-9, a 92kDa gelatinase, is involved in not only in ECM breakdown, but also leukocyte cardiac invasion, cytokine activation, and angiogenesis. MMP-9 and innate immune cells are both required for the development of cardiac rupture in MI [3, 4]. Acetyl-seryl-aspartyl-lysyl-proline (Ac-SDKP) is a naturally occurring acetylated tetrapeptide that exert cardioprotective effects in several cardiovascular and renal diseases when it is exogenously administered [5, 6]. The 43 aminoacid peptide thymosin β4 (Tβ4) carries Ac-SDKP sequence on its N-terminal end and its enzymatic cleavage is the main source of Ac-SDKP. It has been reported that in MI, Tβ4 treatment diseases mortality and cardiac dysfunction concomitant with reduced neutrophil and macrophage infiltration [7]. It is not known whether Ac-SDKP mediates some of the cardioprotective effects of Tβ4. Previous studies showed that the chronic infusion of Ac-SDKP reduces cardiac remodeling in infarcted rats [8]. However, in this study the effects of Ac-SDKP on the prevalence of cardiac rupture and mortality were not evaluated since rats do not develop cardiac rupture. We hypothesized that in the acute stage of MI, Ac-SDKP decreases the incidence of cardiac rupture and mortality by modulating the innate immune response and by preventing the ECM degradation. Furthermore, we evaluated whether Ac-SDKP modulates the differentiation of macrophages towards the pro-inflammatory M1 or reparative/pro-fibrotic M2 phenotypes.

## Material and methods

### Animals

Twelve-week-old male C57BL/6 mice (25–30 g, The Jackson Laboratory, Bar Harbor, ME) were housed in vented cages with a 12:12-h light-dark cycle and given standard chow (0.4% sodium) and tap water. They were allowed 7 days to adjust to their new environment. All protocols were approved by the Institutional Animal Care and Use Committee of Henry Ford Health System.

### Experimental protocols

Mice were anesthetized with pentobarbital sodium (50 mg/kg ip), and an Alzet osmotic minipump (Durect, Cupertino, CA) containing either saline or Ac-SDKP (1.6 mg·kg^−1^·day^−1^) was implanted intraperitoneally at least 48 hours before MI induction. The dose and drug delivery route were based on previous studies of Ac-SDKP [6, 9]. MI was surgically induced by ligating the left anterior descending coronary artery as previously described [7, 10]. To evaluate cardiac rupture incidence and infarct size, treatments were continued for 7 days. The presence of a large amount of blood in the chest cavity and perforation of the infarct-free wall were criteria of death due to cardiac rupture. To evaluate the Ac-SDKP effects on acute inflammation, the animals were studied for 2 days after MI was induced.

### Infarct size

The effect of Ac-SDKP on infarct size was determined at 7 days post-MI as described previously [11]. Briefly, a 6-μm section from each cardiac tissue slice was stained with Gomori Trichrome to identify fibrous tissue (infarction). Infarct size was calculated as a percentage of the infarcted area divided by the entire LV area.

### Immunohistochemistry of cell infiltration

At 24–48 hours post-MI, mice were anesthetized with pentobarbital sodium (50 mg/kg, ip) then the whole heart was perfused with ice-cold PBS for 3 minutes. The heart was sectioned transversely into 3 slices, and the middle section was fixed in formalin. The sections were epitope-retrieved and immunostained with anti-Ly-6g/c antibody, clone: NIMP-R14 (Abcam, Cambridge, MA). Negative controls were processed in a similar fashion, but were not incubated with the primary antibody. All measurements and analyses were performed in a blinded fashion.

### Flow cytometry analysis of immune cells in bone marrow, peripheral blood, and myocardium

Mice were sacrificed at 48 hours post MI. Animals were anesthetized with pentobarbital sodium (50 mg/kg ip). Bone marrow cells from tibias and femurs were collected by flushing the bones with 1 ml PBS. Blood was collected from vena cava, and red blood cells were lysed with ACK lysis buffer. PBS-perfused hearts were enzymatically digested with 0.05% collagenase II (Worthington Biochemical Corporation, Lakewood, NJ), 0.005% elastase (Worthington Biochemical Corporation, Lakewood, NJ), and 10 μg/ml DNase (Sigma Aldrich, St. Louis, MO) for 30 minutes at 37° C. Cells were passed through 70-μm nylon mesh and separated by percoll centrifugation. Cells were blocked with anti-CD16/32 antibody for 5 minutes, then immunostained with anti-mouse CD45 PercP-Cy5.5, anti-mouse CD11b Alexa fluor 700, anti-mouse F4/80 Alexa fluor 488, anti-mouse Ly-6g APC-Cy7, and anti-mouse CD206 PE antibodies. All antibodies were purchased from Biolegend (San Diego, CA). To exclude dead cells, LIVE/DEAD® Fixable Aqua Dead Cell Stain Kit was used (Invitrogen, Carlsbad, CA). Count Bright Absolute Counting Beads (Invitrogen, Carlsbad, CA) were used to calculate the results as the absolute infiltrating cell number per whole tissue (heart). Data were acquired using a BD Fortessa flow cytometer then neutrophils, total macrophages, and M1/M2 macrophage phenotypes were analyzed as described by Yan X et al.[12].

### *In vitro* neutrophil chemotaxis assessment

Cell chemotaxis was evaluated by transwell migration assay using the differentiated HL-60 myeloid cell line purchased from American Type Culture Collection (ATCC, Manassas, VA). Cells were grown in Iscove’s modified Dulbecco’s medium (IMDM) containing 10% fetal bovine serum. HL-60 cells were differentiated into neutrophil-like cells by adding 1.3% DMSO in IMDM. Cells were incubated with vehicle, Ac-SDKP (100 nM), or Tβ4 (20 nM) as a positive control for 15 minutes before the chemotaxis assay. 0.5 million cells were placed in the upper transwell chamber, and a chemoattractant peptide, fMLP (1 nM), was added into the bottom chamber. After 3 hours of incubation, migrated cells were quantified in the bottom chamber using a Neubauer hemocytometer.

### Cardiac myeloperoxidase activity

Frozen heart samples (20–50 mg) were homogenized in 0.5 ml phosphate buffered saline using a dounce homogenizer. Samples were sonicated and centrifuged at 10,000 x g for 10 min at 4°C. Protein concentration in the supernatants was determined by the Bradford method. Samples (0.5 mg of total protein) were concentrated with Amicon Ultra 30K centrifugal filters. We used the myeloperoxidase chlorination fluorometric assay kit (Cayman Chemical) to determine MPO activity following the manufacturer’s protocol. MPO was expressed as relative fluorescence units (RFU) per minute per mg protein.

### Zymogram

Freshly isolated heart tissues were homogenized in ice-cold extraction buffer containing 10 mM cacolydic acid, 0.15 M NaCl, 20 mM ZnCl_2_, 1.5 mM NaN_3_, 1% Triton X-100 (pH 5.0) with a proteinase inhibitor cocktail set III (EMD Millipore). Samples were centrifuged at 14,000 x g for 10 min at 4° C, and protein concentration was determined by Bradford method in the supernatants. Twenty micrograms of protein were loaded in 10% zymographic gel (Invitrogen). Gels were incubated with renaturing buffer (2.5% triton X-100) at room temperature for 30 minutes. Gels were incubated in developing buffer (50 mM Tris-HCl pH 8.0, 5 mM CaCl_2_, 0.2 NaCl, 0.02% Brij-35) for 16 hours at 37° C. Gels were stained in 0.05% coomasie blue solution, then washed with destaining buffer (5% acetic acid, 10% methanol) until sharp bands were visible. Gels were scanned, and bands density was analyzed with Image J software.

### ELISA

Freshly isolated heart tissues were homogenized in ice-cold extraction buffer containing 20 mM HEPES pH 7.5, 2 mM EDTA, 1.5 M NaCl, complete mini EDTA-free proteinase inhibitor and phosSTOP phosphatase inhibitor (Roche) using a dounce homogenizer. Samples were sonicated and centrifuged at 10,000 x g for 10 min at 4°C. Protein concentration in the supernatants was determined by the Bradford method. IL-1β and the specific endogenous tissue inhibitor of metalloproteinases, TIMP-1, were measured in samples (0.5 mg of total protein) using ELISA kits from R&D systems.

### Statistical analysis

Binary data (cardiac rupture and mortality) were expressed as proportions, and groups were compared using a chi-squared test. For cardiac rupture and mortality we proposed to sample a total of 100 animals. This would have resulted in the paired t-test having 80% power, with a two-sided 0.05 alpha level to detect a small effect size of 0.28. In this setting, the effect size represents the detectable difference in standard deviation units. If we assume a high satisfaction rate of 80% then the sample size results in a confidence interval half-width of 8%.

Continuous data are expressed as means ± SE. The data violated the assumptions of the parametric test so we utilized a nonparametric test in its place. An adjustment on the significance criteria is made, using Hochberg’s approach, which is a reasonable approach. An overall test, such as Kruskal-Wallis test, was not utilized since these overall tests are not informative as we are interested in pairwise contrasts and not a global hypothesis. It is a reasonable approach to just examine the pairwise contrasts as long as an adjustment is made on the rejection criteria. Adjusted P values of < 0.05 were considered significant. The sample size was determined based on our previous experience. Groups of n = 10 are appropriate for the physiological experiments to ensure a minimum of 8 mice per group complete the protocols. Based on our previous experience, n = 8 provided sufficient power (80% or higher) to detect small changes in the parameters measured in this study with a P-value of 0.05 or less.

All statistical analysis were performed by the Biostatistics core at the Department of Public Health Sciences, Henry Ford Hospital, Detroit, Michigan.

## Results

### Effect of Ac-SDKP on cardiac rupture and mortality after MI

**Fig 1:**
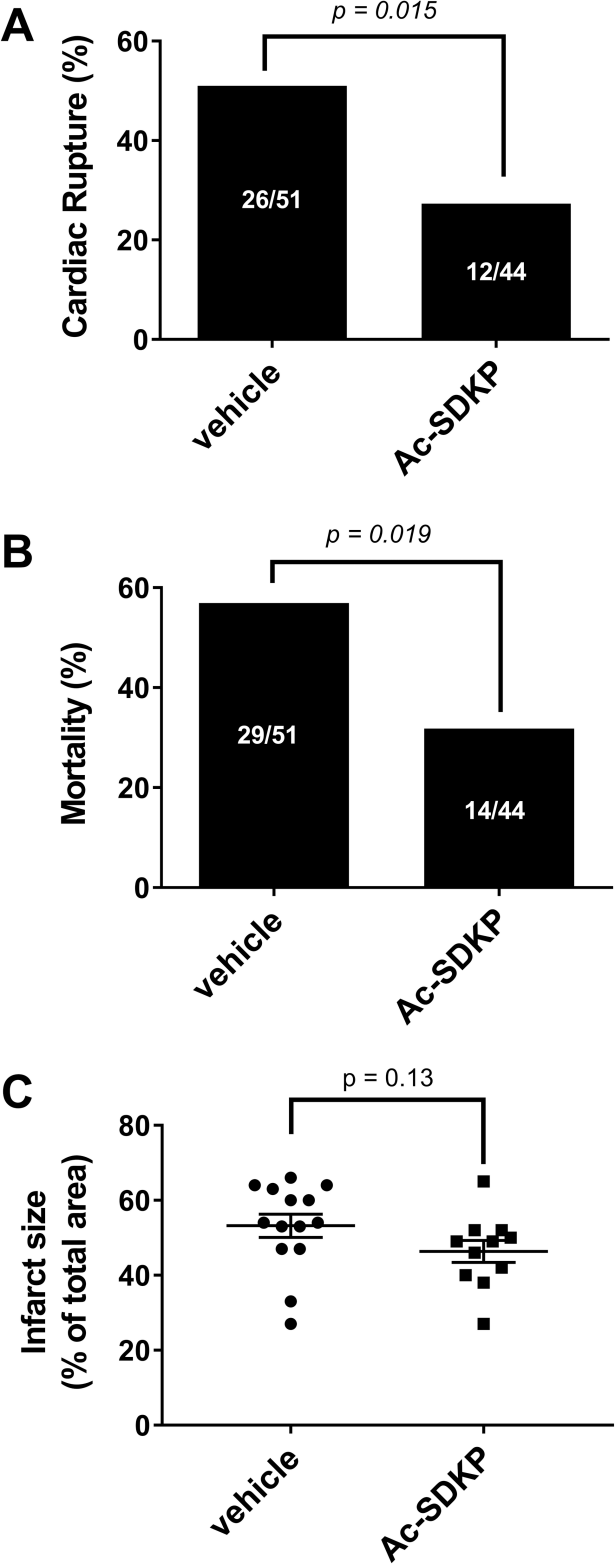
Effect of Ac-SDKP on cardiac rupture incidence, mortality rate, and infarct size in myocardial infarction. Out of 95 mice analyzed at 7 days post-MI, 38 died from cardiac rupture, and 5 died from other causes. In myocardial infarction, Ac-SDKP decreased A) incidence of cardiac rupture from 51.0% (vehicle treated) to 27.3% (Ac-SDKP treated) and B) mortality from 56.9% (vehicle treated) to 31.8% (Ac-SDKP treated). C) In a new group of 25 animals, Ac-SDKP tends to decrease infarct size, but it does not reach statistical significance (p = 0.13; power < 0.70).

MI was performed in 109 mice, 14 animals (12.8%) were excluded from analysis: 6 animals because died during surgery and 8 animals because of not having MI on autopsy. Out of 95 mice analyzed at 7 days post-MI, 38 of those died from cardiac rupture and 5 died from other causes. In vehicle treatment group, the mortality rate was 56.9% and the prevalence of cardiac rupture was 51.0% (26 out of 51 animals). In Ac-SDKP treated animals the prevalence of cardiac rupture (27.3%, 12 out of 44 animals, p = 0.015, Fig 1) and the mortality rate (31.8%, p = 0.019, Fig 1B) was significantly lower in comparison to vehicle treated animals. In a different cohort of 25 animals we tested the effects of Ac-SDKP on infarct size. Although there was not difference between the vehicle vs Ac-SDKP treated animals, there is a trend that Ac-SDKP tends to decrease the infarct size (p = 0.13, power of test < 0.07; Fig 1C).

### Effect of Ac-SDKP in myocardial inflammatory cell infiltration after acute MI

**Fig 2:**
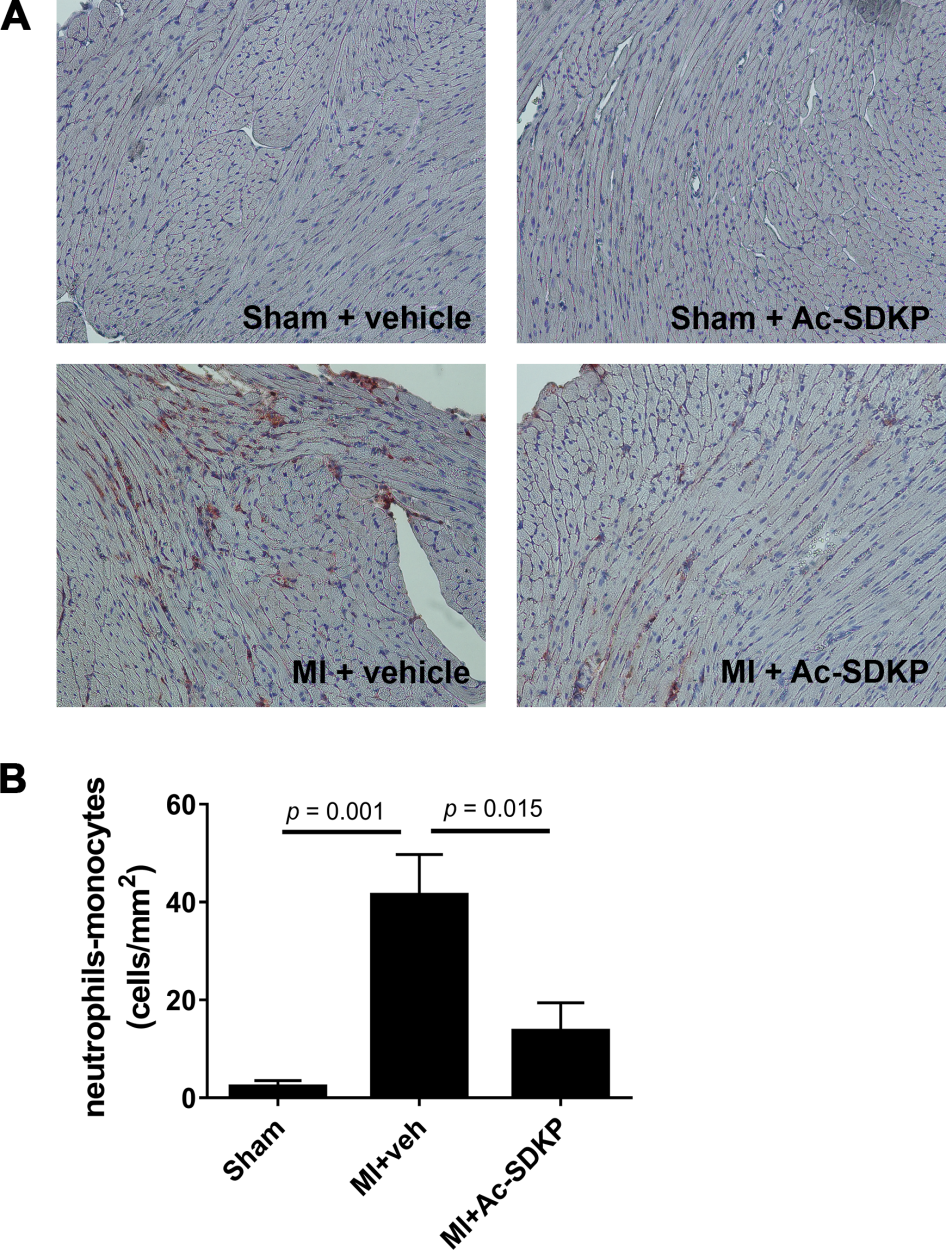
Effects of Ac-SDKP on cardiac inflammatory cell infiltration in myocardial infarction. A) Representative photoraphs of myocardial sections stained for Ly-6C/G (neutrophil/monocyte marker) from sham and 24 h post-myocardial infarction (MI). Ac-SDKP infusion started 48 h before surgery. Magnification was 20x. Only Ly-6g/c positive (*brown*) cells with visible nuclei were counted. Photographs of the whole section surface were taken. Analysis was performed blinded. B) Quantitative data of myocardial Ly-6C/G positive cells expressed as cells per mm^2^. In MI, Ac-SDKP blunted neutrophil/monocyte infiltration.

**Fig 3:**
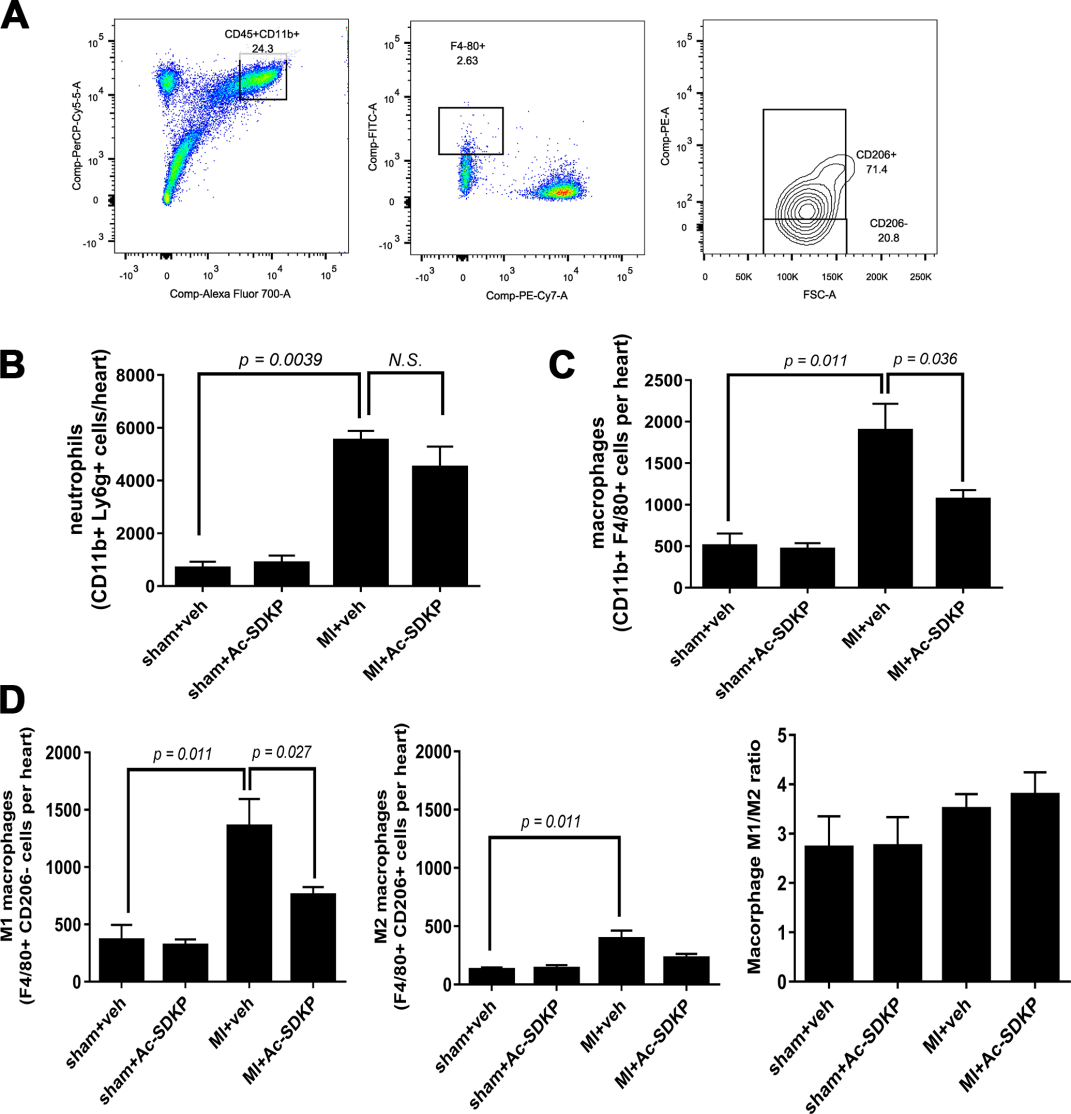
Effects of Ac-SDKP on cardiac neutrophils and macrophages in myocardial infarction. Cardiac infiltration by neutrophils and macrophages was analyzed by flow cytometry at 2 days post-myocardial infarction (MI). A) Gating strategy: After gating for live cells using viability dye, total myeloid cells (CD45^+^ CD11b^+^) were gated. Macrophages were identified as F4/80^+^ Ly^-^6g^-^ cells whereas neutrophils were identified as F4/80^-^ Ly^-^6g^+^ cells. Using Count Bright Absolute Counting Beads (Invitrogen, Carlsbad, CA) total neutrophils (B) and macrophages (C) per heart were quantified. Ac-SDKP significantly reduced expansion of cardiac macrophage but not neutrophil populations in MI. The pro-inflammatory M1 and pro-fibrotic/reparative M2 polarization (D) was analyzed in the total macrophage (F4/80^+^) gate. Both, M1 (CD206^-^) and M2 (CD206^+^) were increased in MI. Ac-SDKP prevented the increase in M1 macrophages and tended to decrease M2 macrophages.

Immunohistochemical analysis of cardiac tissues after 24–48 hours post MI using anti-Ly-6g/c antibody, a myeloid cell derived marker, revealed a prominent myocardial infiltration by immune cells. Infusion of Ac-SDKP from 3 days before the surgery prevented the infiltration by immune cells in MI (Fig 2A and 2B). Since Anti-Ly-6g/c antibody recognizes both neutrophils and monocytes in a separate cohort of mice we performed flow cytometry analysis to identify the phenotype of these cells (Fig 3A). Using cell specific antibodies we observed that in MI both neutrophils and monocytes were increased. Pre-treatment with Ac-SDKP did not affect neutrophil infiltration (Fig 3B) but it reduced the number of macrophages (Fig 3C). During the acute stage of MI (24–48 hours) the M1 pro-inflammatory phenotype was dominant over M2 anti-inflammatory macrophages. Ac-SDKP decreased M1 macrophages but it did not affect M2 macrophages. No changes in the M1/M2 ratio was observed (Fig 3D). The treatment with Ac-SDKP had no effect in sham operated animals.

### Effect of Ac-SDKP in myeloid derived cells in bone marrow and peripheral blood after acute MI

**Fig 4:**
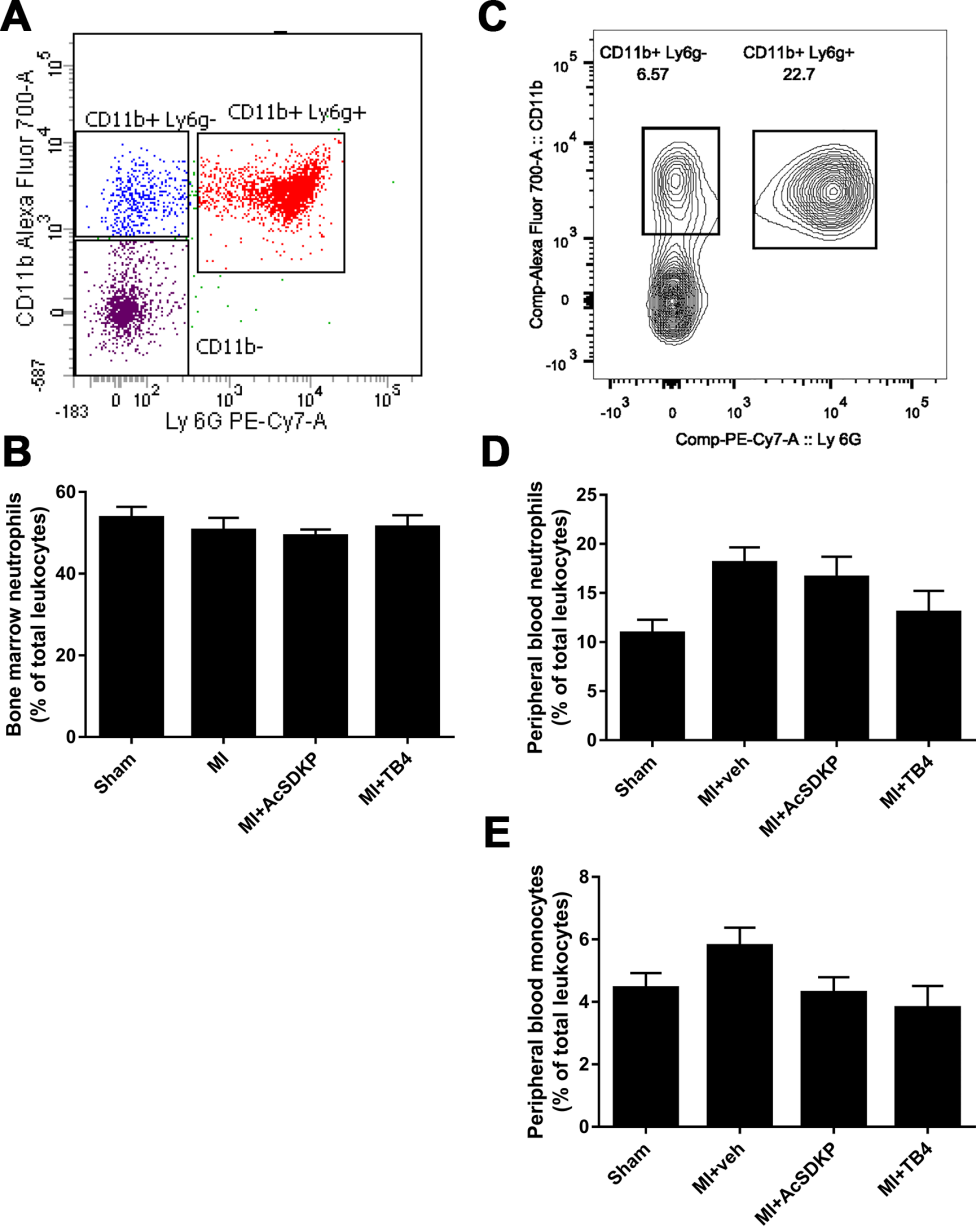
Evaluation of innate immune cells in bone marrow and peripheral blood by flow cytometry analysis. A) Representative flow cytometry analysis of bone marrow. Neutrophils were identified as CD11b^+^Ly6g^+^. B). Ac-SDPK and thymosin β (Tβ4) do not affect bone marrow neutrophil mobilization in myocardial infarction (MI). C) Representative flow cytometry analysis of peripheral blood. Neutrophils were identified as CD11b^+^ Ly6g^+^ and monocytes were CD11b^+^ Ly6g^-^. MI induced increased peripheral blood neutrophils (D), but did not affect circulating monocytes (E). Tβ4 tends to decrease both neutrophils and monocytes, whereas Ac-SDKP tends to decrease monocytes (these differences were not statistically significant).

The flow cytometry analysis showed not differences in neutrophil population in bone marrow after the MI (Fig 4A and 4B). However, in peripheral blood we observed that myocardial infarction (MI) induced an increase in neutrophils, that was not restored by neither Ac-SDKP or Tβ4 infusion (Fig 4D). Peripheral blood monocytes (Ly6g^-^) tend to increase after the MI, and both Ac-SDKP and Tβ4 tended to restore the amount monocytes after MI, however these differences were not statistically significant (Fig 4E).

### Effect of Ac-SDKP and Tβ4 on neutrophil chemotaxis

**Fig 5:**
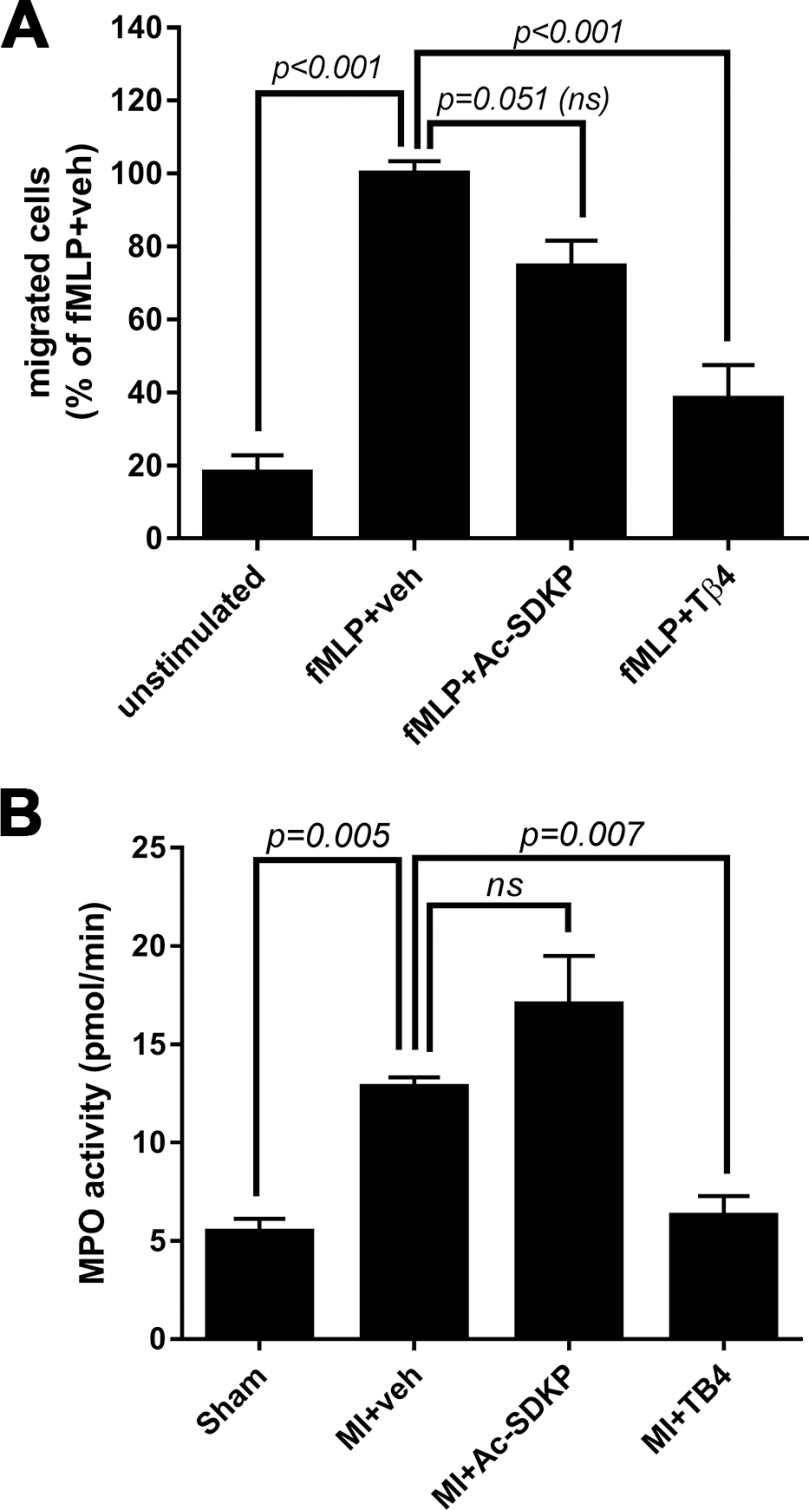
Effects of Ac-SDKP and thymosin β (Tβ4) on neutrophils *in vitro* and *in vivo*. A) Transwell assays were conducted to evaluate the effect of Ac-SDKP and Tβ4 on fMLP-induced neutrophil (HL-60 cell line) chemotaxis *in vitro*. Tβ4, but not Ac-SDKP, inhibited fMLP-induced neutrophil chemotaxis. B) cardiac myeloperoxidase (MPO) activity was measured at 48 hours post-myocardial infarction (MI). Tβ4, but not Ac-SDKP, prevented MPO activation in MI.

The effect of Ac-SDKP and Tβ4 on neutrophil chemotaxis was assessed *in vitro* by transwell chemotaxis assay. As shown in Fig 5A we observed that Tβ4, but not Ac-SDKP, inhibited neutrophil migration induced by the formylated peptide fMLP.

### Effect of Ac-SDKP on myeloperoxidase (MPO) and MMP-9 in MI

We tested the effect of Ac-SDKP and Tβ4 at a dose of 1.6 mg/kg/day on myocardial MPO activity at 24 hours post MI. As shown in Fig 5B, MPO activity was increased in MI in comparison to sham operated animals. Tβ4, but not Ac-SDKP, prevented the increase in MPO activity in infarcted hearts.

### Zymography

**Fig 6:**
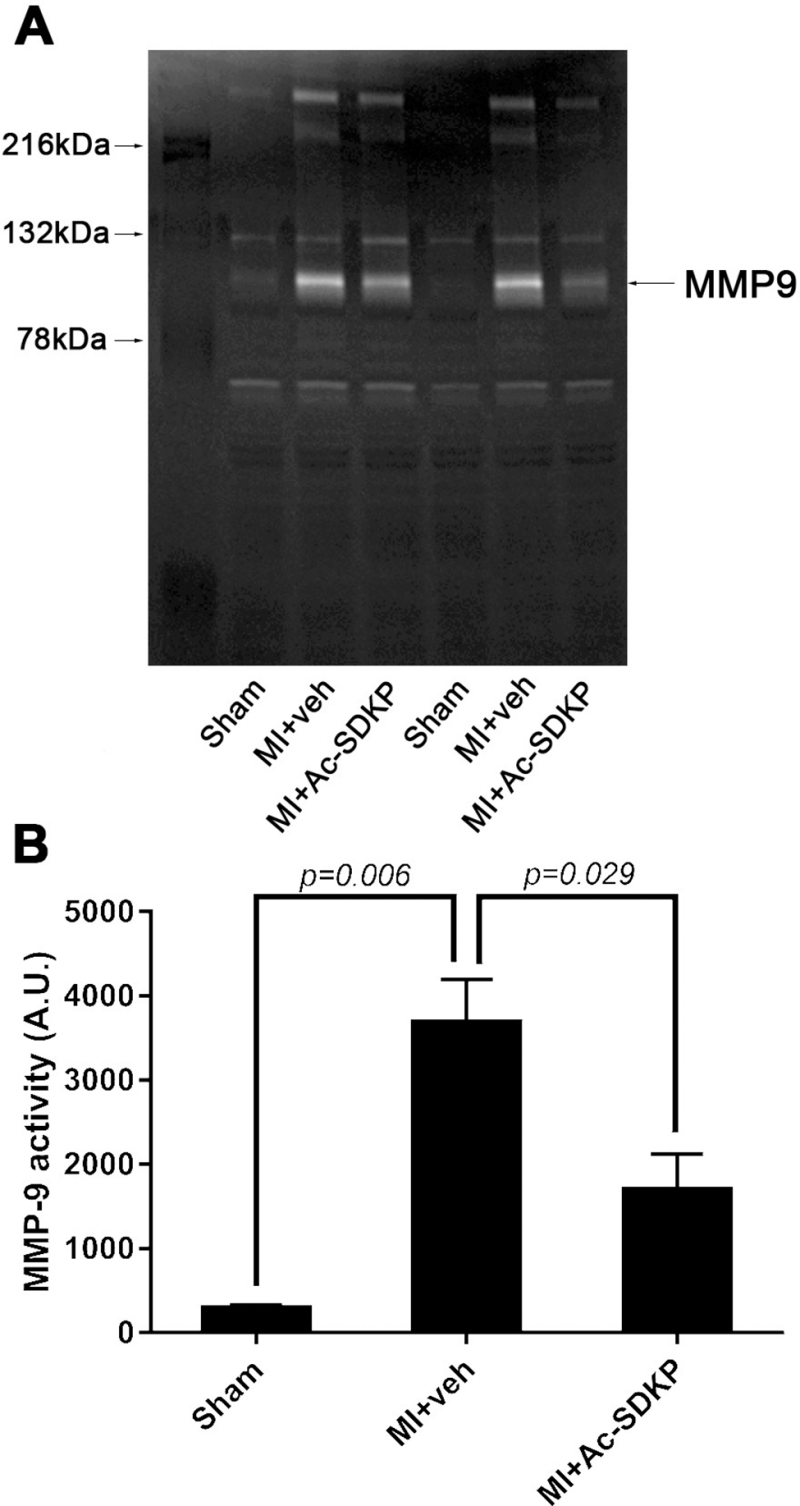
Effects of Ac-SDKP on cardiac matrix metalloproteinase (MMP) activity in myocardial infartion. Ac-SDKP effects on MMP activity were measure by zymography A) Representative figure of zymograms. *White bands* represent degradation of gelatin matrix, indicating enzymatic activity. Myocardial infarction activated MMP-9 (~ 92 kDa protein) but the band corresponding to MMP-2 activity (~ 62 kDa protein) was not observed (confirmed using a positive control, data not shown). Ac-SDKP significantly decreased MMP-9 activity in infarcted animals. B) Quantitative data were obtained by measuring band optical intensity.

An increase in MMP-9 levels were observed in infarcted mice at 48 hours post-MI, and Ac-SDKP diminished this increase. No significant alterations in MMP-2 activity was observed in either sham or MI groups (Fig 6A and 6B).

### Effect of Ac-SDKP on cardiac TIMP-1 and IL-1β

**Fig 7:**
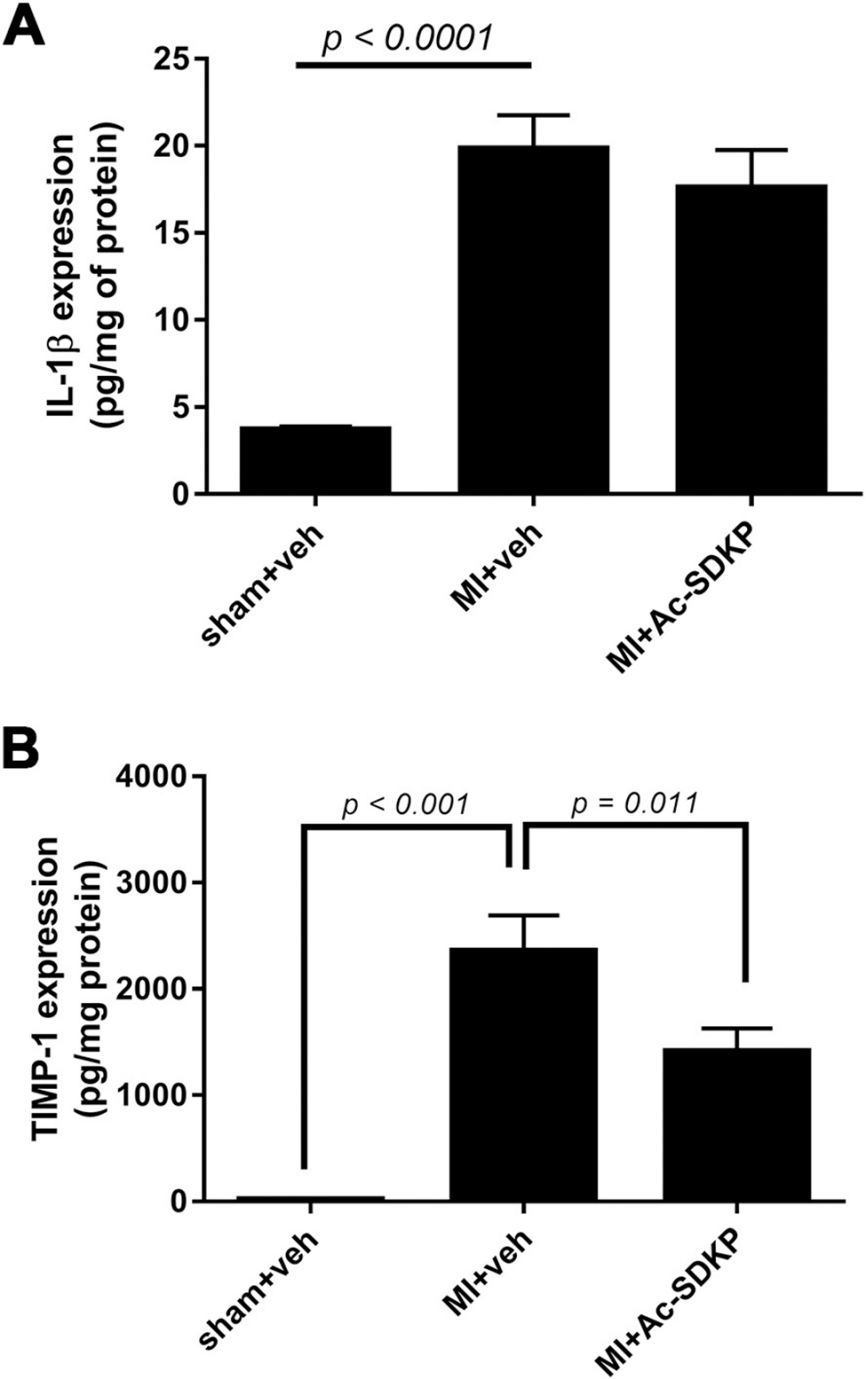
Effects of Ac-SDKP on cardiac interleukin 1β (IL-1β) and endogenous tissue inhibitors of metalloproteinases (TIMP-1). A) IL-1β was measured by ELISA at 48 hours post-myocardial infarction (MI). There was a significant increase in IL-1β expression in MI. Ac-SDKP did not prevent increased in IL-1β. B) Measurement of TIMP-1 at 48 hours post-MI by ELISA indicated that the increase in TIMP-1 in MI is reduced by Ac-SDKP.

The specific endogenous tissue inhibitor metalloproteinase TIMP-1 and IL-1β were measured by ELISA at 48 hours post-MI. Both, TIMP-1 and IL-1β were significantly increased in MI. Ac-SDKP reduced TIMP-1 but has not effect on IL-1β in infarcted animals (Fig 7A and 7B).

## Discussion

The present study aims to describe the effects of Ac-SDKP during the acute stage of MI. ACE inhibitors are one of the most effective drugs to alleviate cardiac inflammation and remodeling in MI. Ac-SDKP is a compelling peptide to treat cardiovascular diseases since its endogenous plasma levels increase with ACE inhibitors. Although ACE inhibitors mainly target the renin-angiotensin system, there is evidence indicating that Ac-SDKP is required for ACE inhibitors cardioprotective effects. We and others previously reported that Ac-SDKP inhibits interstitial collagen deposition [13, 14]. Because collagen deposition is required for maintaining structural integrity of the infarcted ventricle [15] we predicted that Ac-SDKP could exacerbate cardiac rupture in mice. On the other hand, anti-inflammatory properties of Ac-SDKP predicts significant benefits in MI. Mice treated with Ac-SKDP and subjected to MI showed a reduced cardiac rupture and mortality compared with vehicle treated mice. Cardiac repair after MI results from several events initiated by neutrophils and macrophages (inflammatory phase) that serves to clear damaged cells and extracellular matrix tissue (3–4 d in mice), followed by a reparative phase with resolution of inflammation, scar formation, and neovascularization over the next several days [15, 16]. Depletion of neutrophils either with hydroxycarbamide or genetic removal of MPO completely prevents cardiac rupture [3, 4]. Tβ4 is an endogenous 43 amino acid peptide containing Ac-SDKP sequence in its N-terminal end. The enzymatic hydrolysis of Tβ4 by meprin and prolyl oligopeptidase releases Ac-SDKP. Previously, Peng et al. demonstrated that Tβ4 prevents cardiac rupture in MI [7] and in the present study we observed that Tβ4 suppresses neutrophil mobilization *in vivo* and *in vitro*. We initially hypothesized that Ac-SDKP may mediate the effects of Tβ4 on neutrophils, but conversely we observed that Ac-SDKP did not decrease neutrophil chemotaxis nor MPO activity, indicating that Ac-SDKP might not be involved in Tβ4 effects at least on neutrophil function. This is important because Tβ4 cleavage releases different peptides besides Ac-SDKP, such as AGES [17], and dissecting the effects of each Tβ4 fragment will be crucial for designing new therapeutic compounds with specific actions. During the acute ischemic event, macrophage expansion occurs, likely through both local macrophage proliferation and circulating monocyte recruitment [18]. Infiltrated macrophages exhibit biphasic activation after MI; pro-inflammatory M1 macrophages peak at MI 3 days, whereas M2 macrophage (pro-fibrotic/reparative) peak at MI 7 days [19]. We found a massive expansion of M1 macrophages at 48 hours post-MI and this increase was suppressed by Ac-SKDP. These observations are not surprising since Sharma et al showed that Ac-SDKP inhibits macrophage differentiation, mobilization, and TNF-α release *in vitro* [20]. M2 macrophages play an important role in the tissue repair after cardiac ischemic injury [21]. Ac-SDKP analog, Ac-S_D_DK_D_P, improves cardiac function but paradoxically attenuates M2 macrophages [22]. Similarly, we observed that Ac-SDKP tended to decrease M2 pro-reparative macrophages. We speculate that the Ac-SDKP decrease in M2 response could be led by the attenuation of inflammatory environment, that is necessary for the M2 polarization. MMP are capable of degrading cardiac extracellular matrix [2]. Mice deficient in MMP-9 are protected from cardiac rupture (Heymans et al., 1999), indicating that MMP-9 plays a role in early myocardial healing. We observed that MMP-9 was activated during the acute phase of MI, and this increase was blunted when the mice were treated with Ac-SDKP. Thus, Ac-SDKP might decrease the incidence of cardiac rupture by preventing the degradation of the extracellular matrix. MMP-9 is released by neutrophils and macrophages, and reduced MMP-9 levels could result from decreased immune cell infiltration. However, MMP-9 also contributes to immune cell infiltration, indicating that there is a positive loop between them. One can argue that Ac-SDKP might be detrimental at the chronic stage of MI because MMP-9 deletion prevents cardiac rupture in the short term, but also inhibits angiogenesis in the reparative phase [3]. Interestingly, long term effects of Ac-SDKP include pro-angiogenic effects [23] and improvement of cardiac function [5, 8]. Ac-SDKP exerts multiple effects in various inflammatory as well as cell repair mechanisms, and the activation/inactivation of all these factors do not occur simultaneously. Thus, MMP inactivation might not necessarily the only mechanism mediating Ac-SDKP effects in MI. TIMP are endogenous inhibitors of MMP-9 and participates in their regulation. Similarly, pro-inflammatory cytokines are positive regulators of MMP-9. Thus, Increased TIMP or decreased pro-inflammatory cytokines could explain how Ac-SDKP interferes with MMP-9 activation. Interestingly, Ac-SDKP did not increased TIMP-1 neither decreased IL-1β. In cardiac fibroblasts Ac-SDKP inhibits IL-1β-induced MMP-9 activity (Rhaleb et al., 2013; Sharma et al., 2008) indicating that Ac-SDKP interferes with the IL-1β receptor signaling. Since Ac-SDKP decreases NF-kB activation *in vitro* and *in vitro* [24, 25] we predict that the inhibition of NF-kB is required for the Ac-SDKP-mediated MMP-9 downregulation. Our experimental design in this study allows us to demonstrate that Ac-SDKP protective effects are observed when treatment is initiated before ligation of the coronary artery. Although a pre-treatment procedure can be interpreted as a limitation, many patients at “high risk” of cardiovascular events are frequently treated with ACE inhibitors. We demonstrated that Ac-SDKP prevents cardiac rupture, a fatal complication without any specific treatment, and that the Ac-SDKP protective mechanisms might involve decreased pro-inflammatory M1 macrophage cardiac infiltration and MMP-9 expression.

## Supporting information

S1 Fig Flow cytometry raw original data supporting the Fig 3C and 3D. Events were acquired using BD Fortessa flow cytometer and analyzed with FlowJo software. Original images of each sample are shown. Quantitative data of the events per sample and per hearts (corrected by the number of counting beads) is shown in the tables.
(PPTX)

S2 Fig Original zymograms supporting the Fig 6. Original zymograms obtained from sham, myocardial infarction + vehicle (MI+veh), and myocardial infarction + Ac-SDKP (MI+Ac-SDKP) heart homogenates.
(PPTX)
