# Supplementary material for: Ac-SDKP decreases mortality and cardiac rupture after acute myocardial infarction
Source: PLoS One. 2018 Jan 24;13(1):e0190300. doi: 10.1371/journal.pone.0190300 (PMC5783348; doi:10.1371/journal.pone.0190300)
Supplement: S1 Fig — Events were acquired using BD Fortessa flow cytometer and analyzed with FlowJo software. Original images of each sample are shown. Quantitative data of the events per sample and per hearts (corrected by the number of counting beads) is shown in the tables. (PPTX) [file pone.0190300.s001.pptx]

## Slide 1
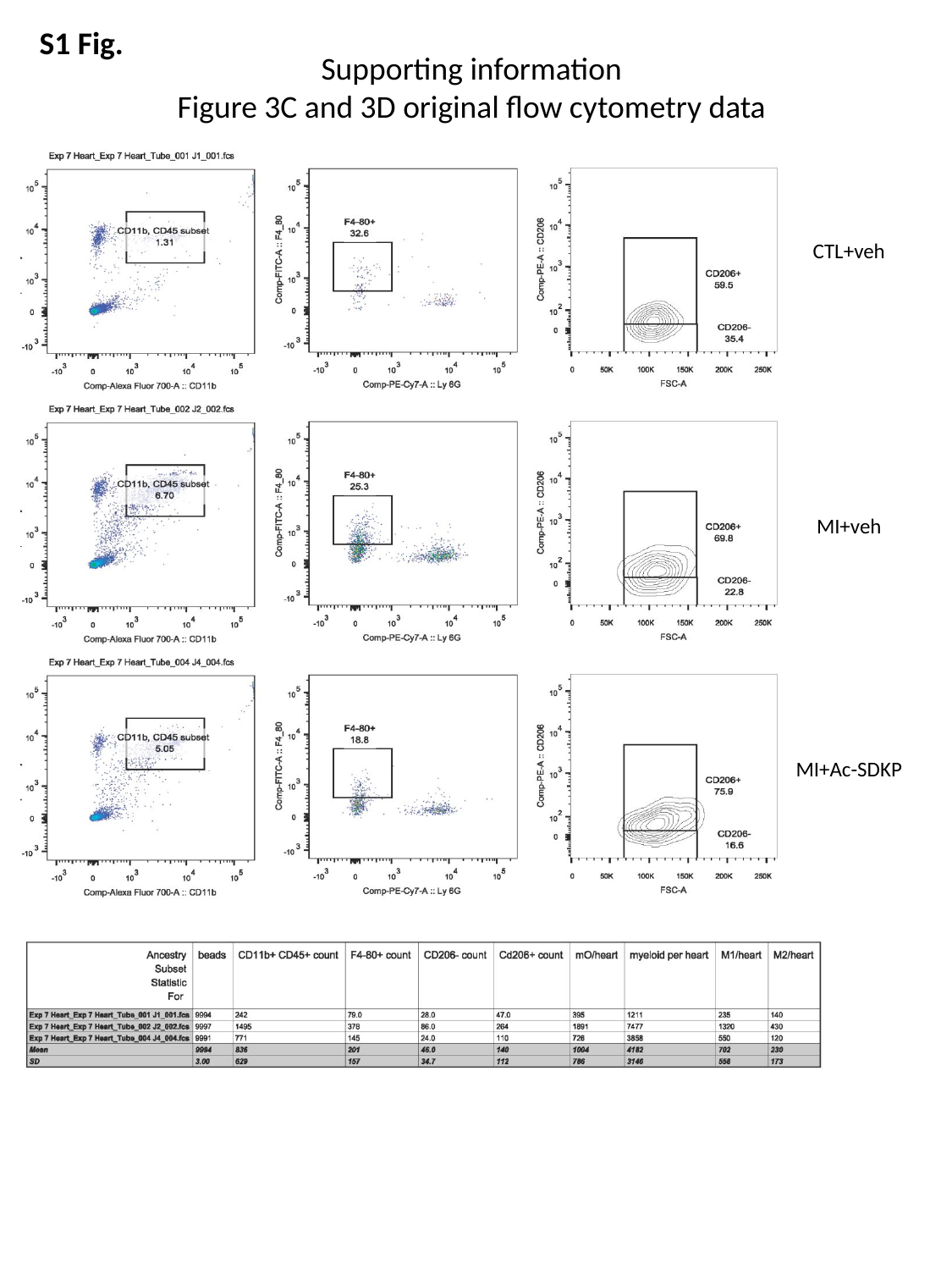

S1 Fig.
Supporting information
Figure 3C and 3D original flow cytometry data
CTL+veh
MI+veh
MI+Ac-SDKP

## Slide 2
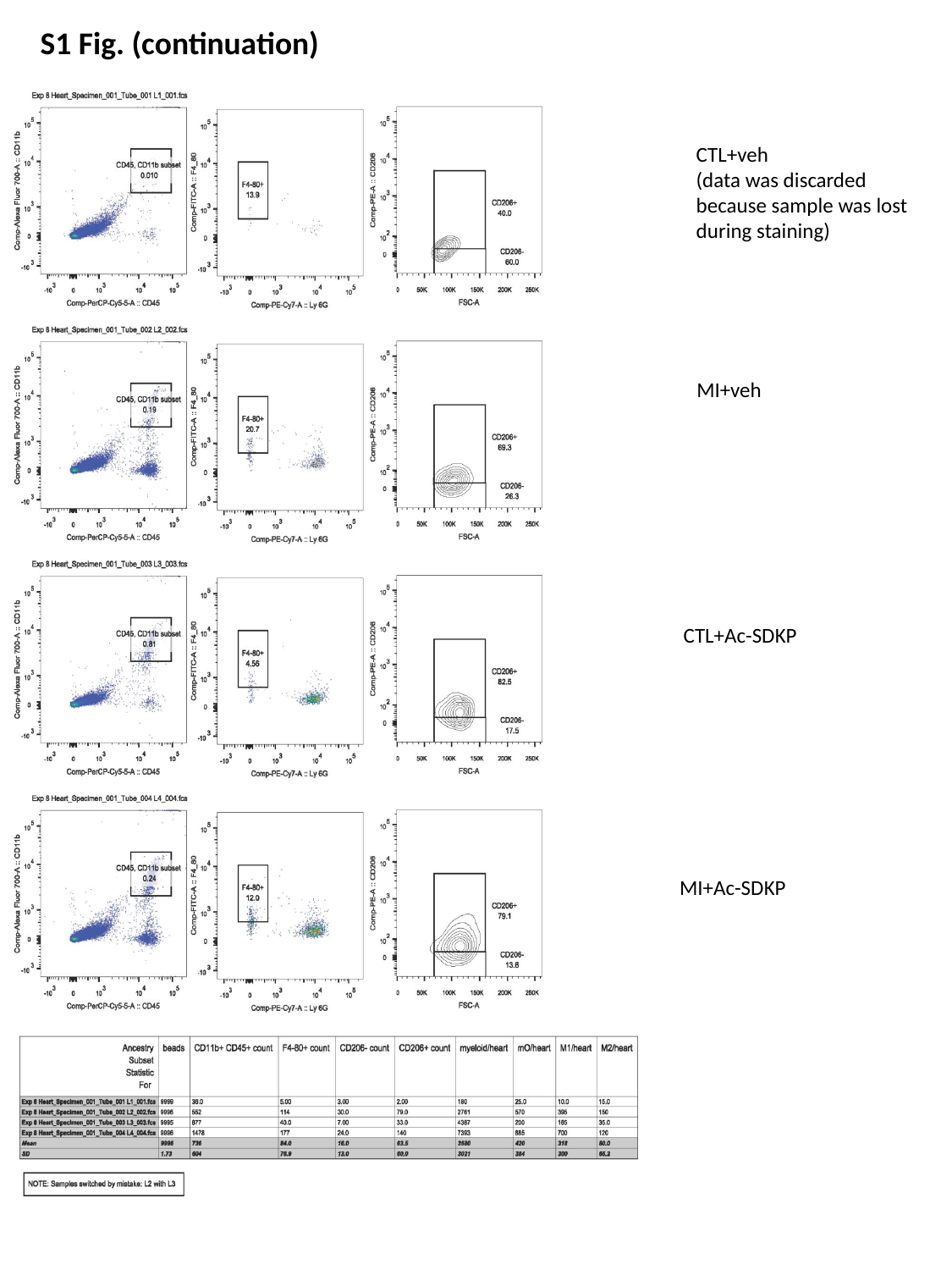

S1 Fig. (continuation)
CTL+veh
(data was discarded because sample was lost during staining)
MI+veh
CTL+Ac-SDKP
MI+Ac-SDKP

## Slide 3
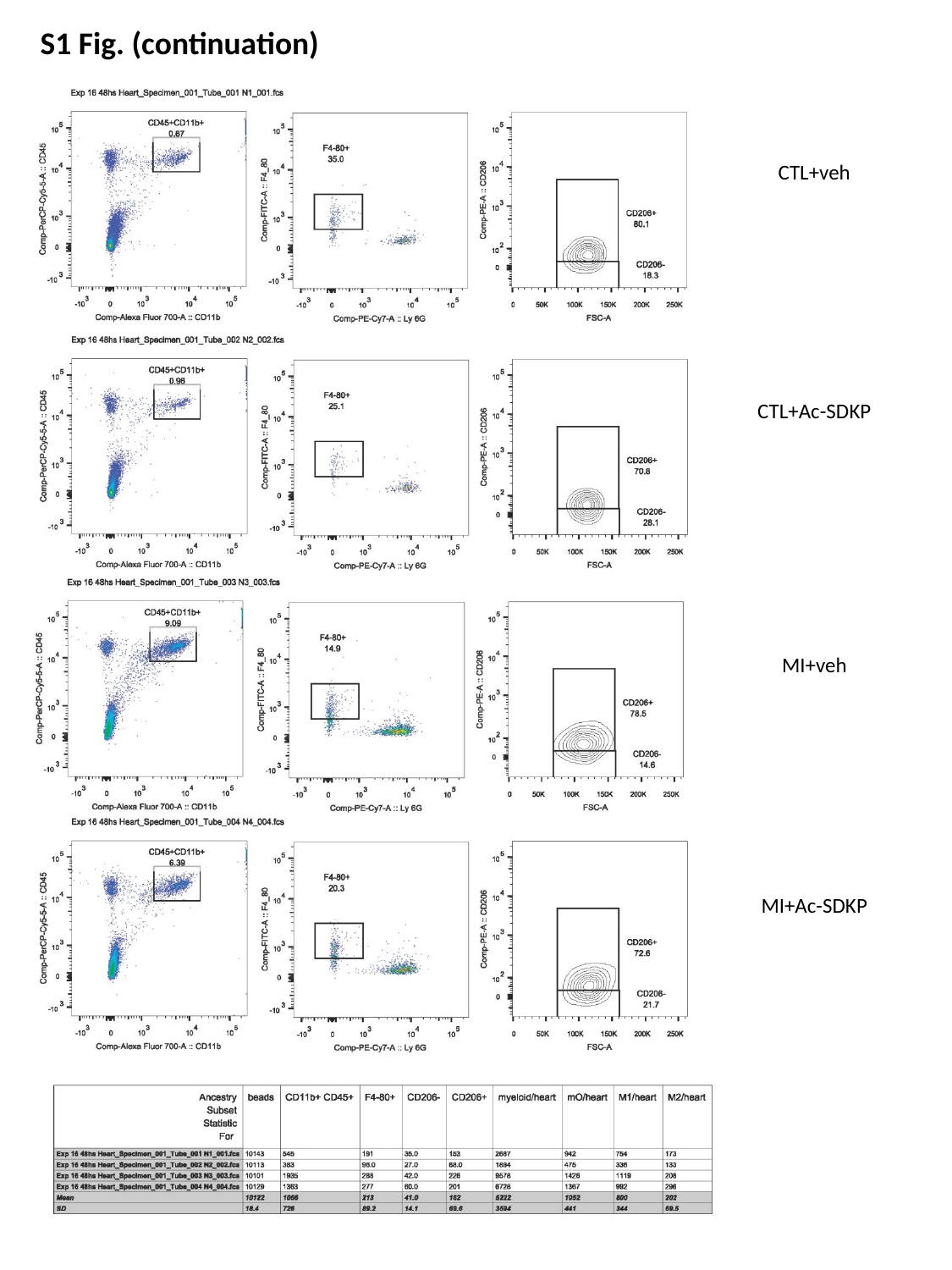

S1 Fig. (continuation)
CTL+veh
CTL+Ac-SDKP
MI+veh
MI+Ac-SDKP

## Slide 4
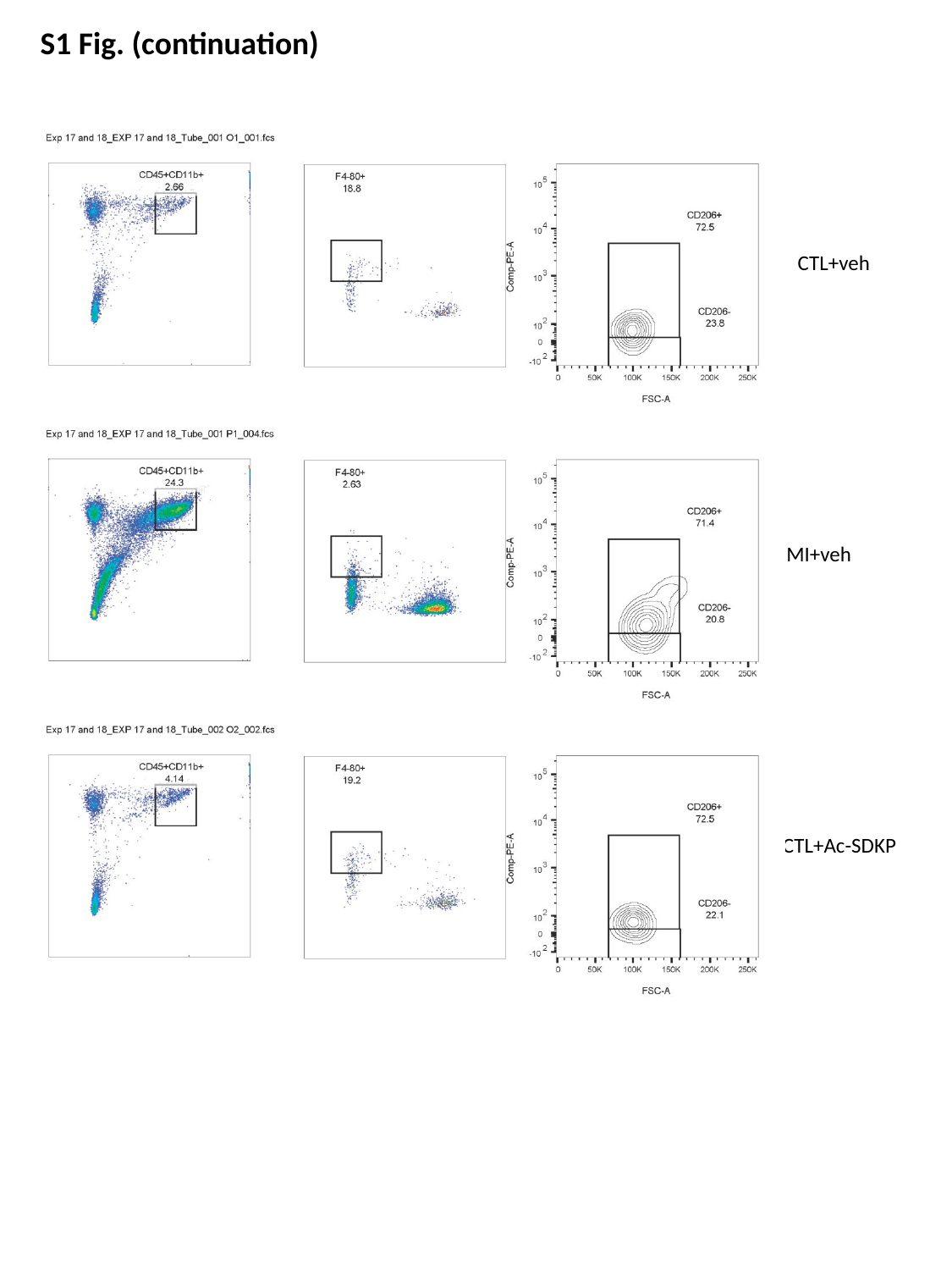

S1 Fig. (continuation)
CTL+veh
MI+veh
CTL+Ac-SDKP

## Slide 5
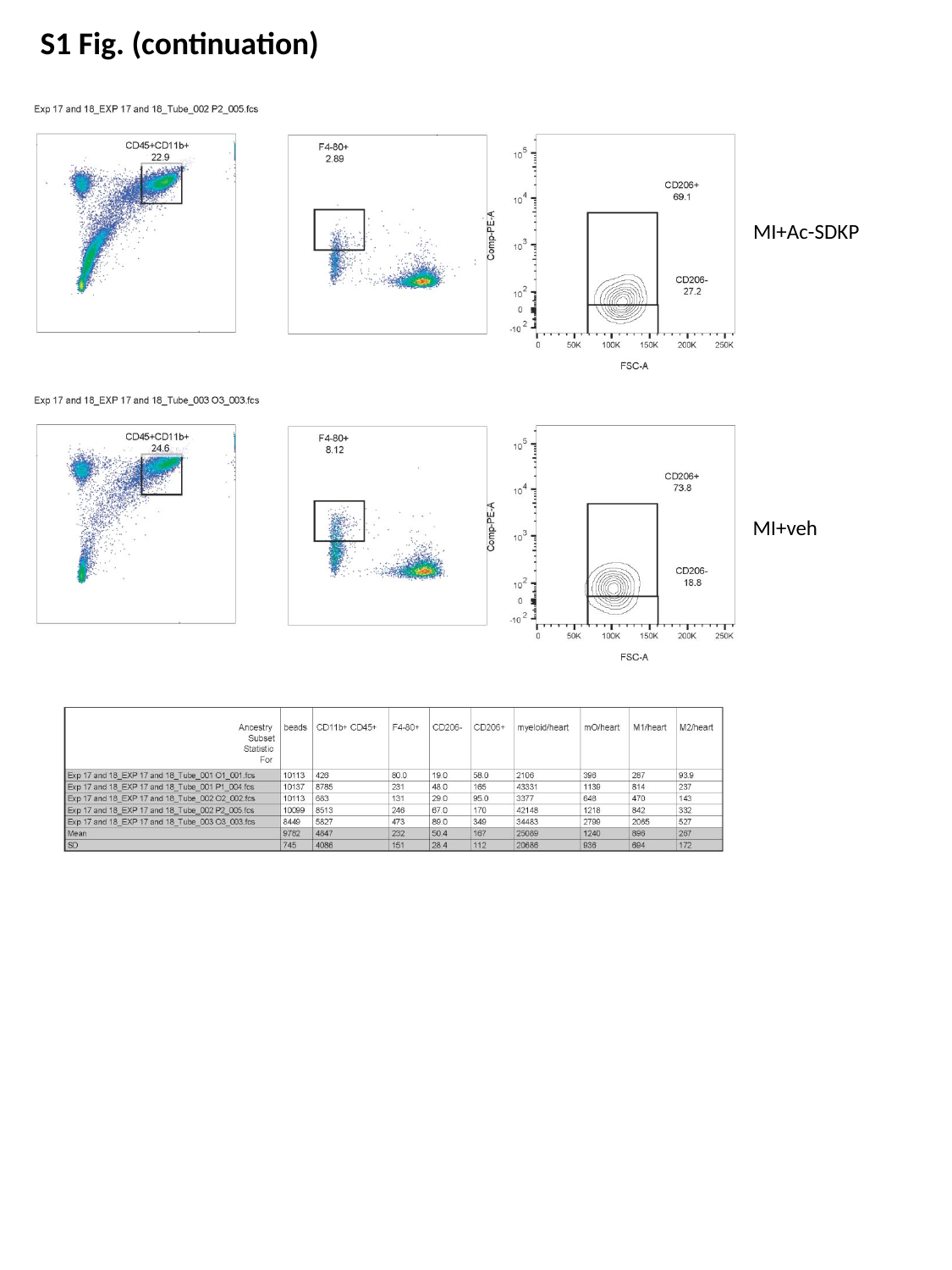

S1 Fig. (continuation)
MI+Ac-SDKP
MI+veh

## Slide 6
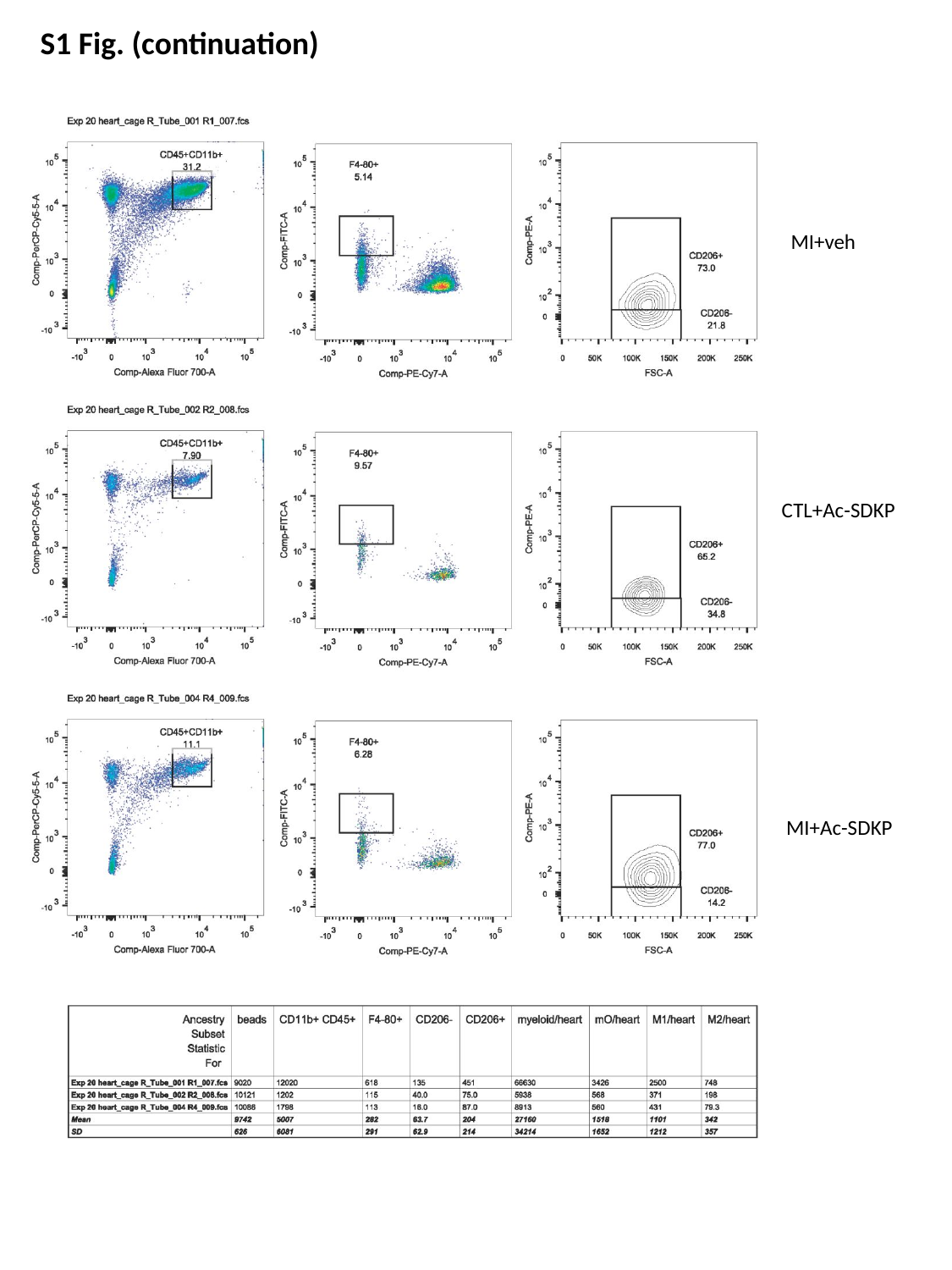

S1 Fig. (continuation)
MI+veh
CTL+Ac-SDKP
MI+Ac-SDKP

## Slide 7
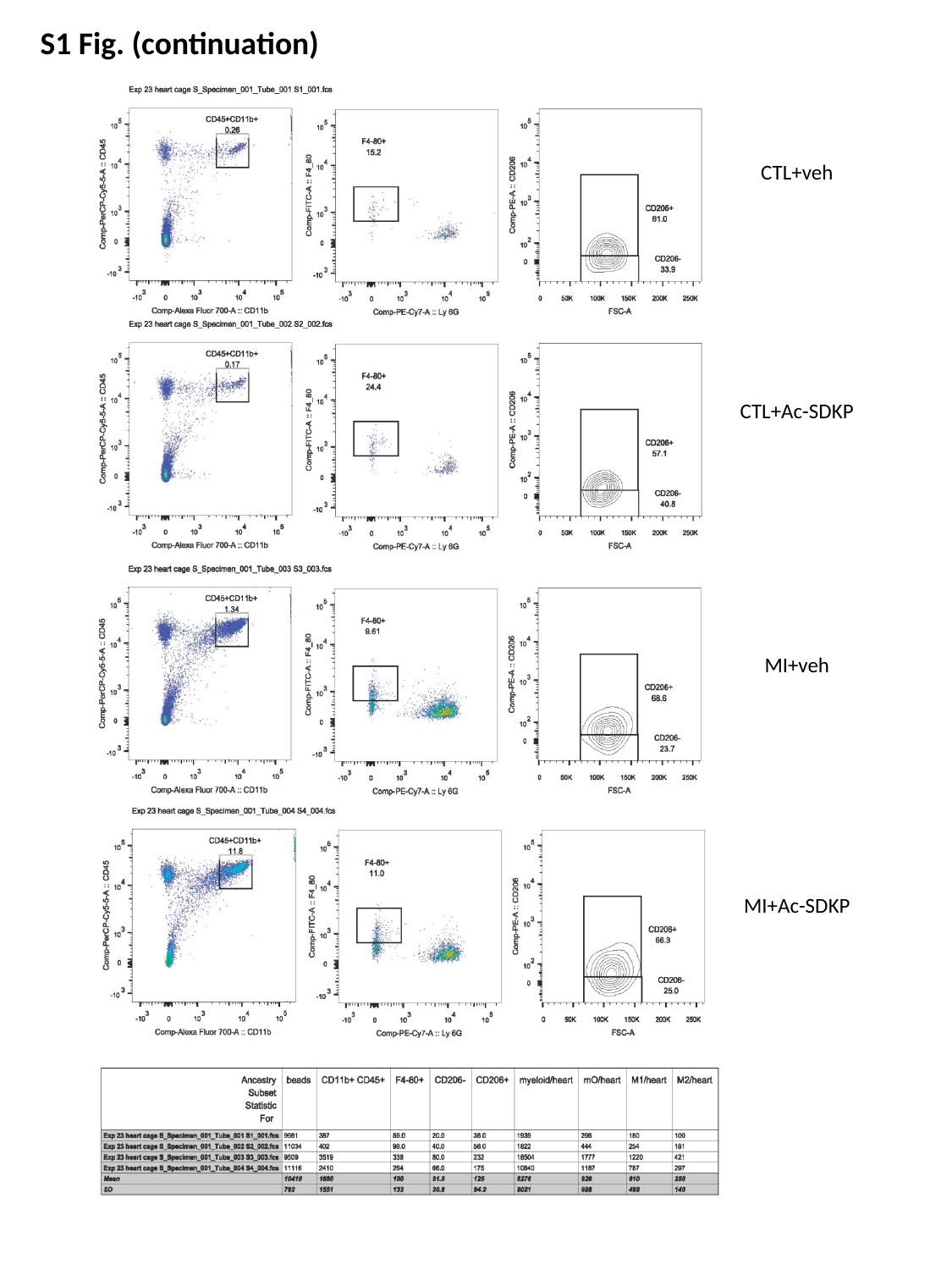

S1 Fig. (continuation)
CTL+veh
CTL+Ac-SDKP
MI+veh
MI+Ac-SDKP

## Slide 8
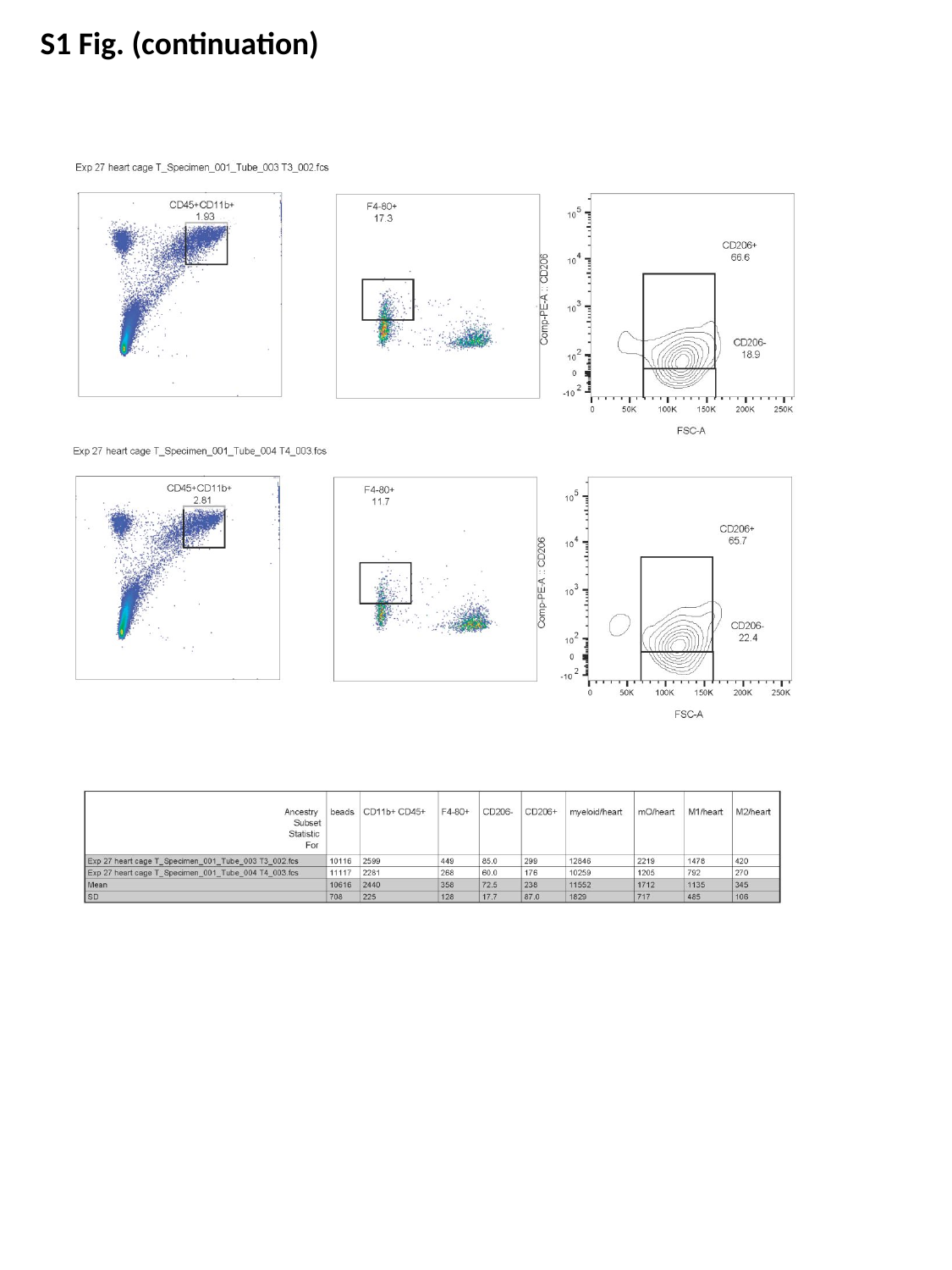

S1 Fig. (continuation)
