# Supplementary figures and images for: Ac-SDKP decreases mortality and cardiac rupture after acute myocardial infarction
Source: PLoS One. 2018 Jan 24;13(1):e0190300. doi: 10.1371/journal.pone.0190300 (PMC5783348; doi:10.1371/journal.pone.0190300)

## Slide 1
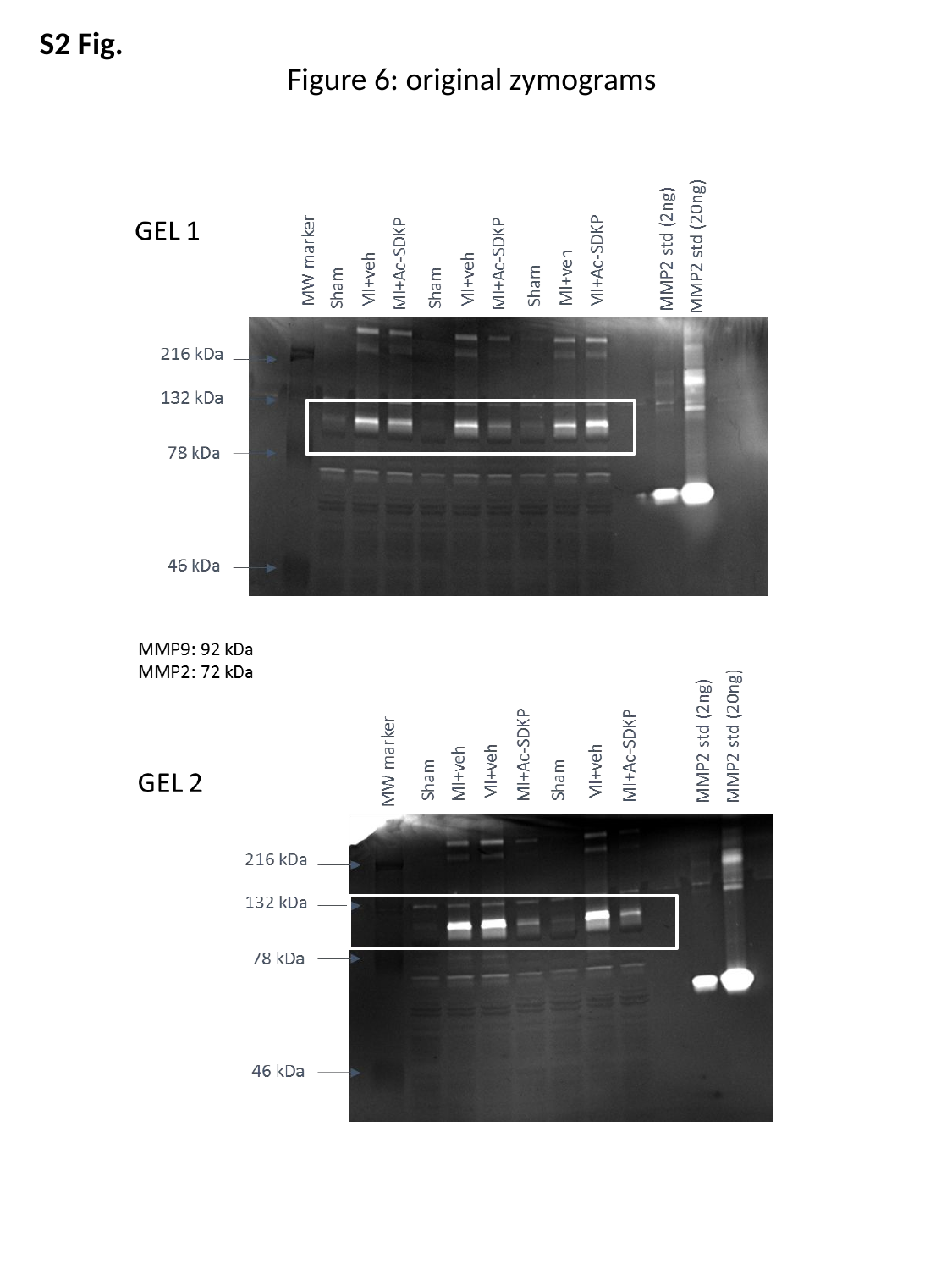

S2 Fig.
Figure 6: original zymograms

Supplement: S2 Fig — Original zymograms obtained from sham, myocardial infarction + vehicle (MI+veh), and myocardial infarction + Ac-SDKP (MI+Ac-SDKP) heart homogenates. (PPTX) [file pone.0190300.s002.pptx]
